# 2388: eResearch suite: A comprehensive platform for electronic consent and data collection

**DOI:** 10.1017/cts.2017.149

**Published:** 2018-05-10

**Authors:** Melissa J. Mueller, Jason Kadrmas

## Abstract

OBJECTIVES/SPECIFIC AIMS: The goal of the eResearch platform is to make consenting for clinical trials more convenient, accessible, and faster while retaining an ethical and informed consenting process. eResearch e-consent also allows for enhanced standardization and efficiency for research collaborations across academic research institutions, which, ultimately, helps drive discovery of better health care for our patients and communities. METHODS/STUDY POPULATION: The UMN’s CTSI and AHC Information Systems developed software, called eResearch Suite, for electronic consenting. The eResearch Suite includes viewing a consent, a “Check Your Understanding” quiz to assess comprehension of critical study details, and a signature block that captures the participant signature electronically and with an automatic date and time stamp. The eResearch Suite also has the capability to randomize participants, track participants via a master list, collect participant data, collect internal study data, and generate emails to participants. The eResearch Suite platform is written in Ruby on Rails. RESULTS/ANTICIPATED RESULTS: We have pilot tested the eResearch platform with one study thus far. Preliminary results of the study show that all participants consented via eResearch, with 64% of participants consenting remotely via eResearch before their first study visit. Participants e-consented using various devices including desktop computers, tablets, and smart phones. Participants also filled out surveys and questionnaires before their study visits, which saved the study team time and money. DISCUSSION/SIGNIFICANCE OF IMPACT: eResearch electronic consenting (e-consenting) changes the way potential participants consent for studies. e-Consenting is important because it allows individuals, or their Legally Authorized Representatives, to consent remotely. This may be faster, more convenient for people, reduce coercion, increase comprehension, and allow for consenting information or process to be shared with an individual’s family/friends. In acute and emergent settings we anticipate eResearch e-consenting will result in significant reduction of consent time by replacing faxed and paper consent with e-consent available via email and mobile devices. This allows legally authorized representatives to sign consent remotely, reduces the time physicians spend faxing consents, and allow them to avert more focus back on their patients. Time savings, whether for consent or study visits, may also result in a cost savings for studies.

